# Opioid-Induced In-Hospital Deaths: A 10-Year Review of Australian Coroners’ Cases Exploring Similarities and Lessons Learnt

**DOI:** 10.3390/pharmacy9020101

**Published:** 2021-05-07

**Authors:** Nicholas Smoker, Ben Kirsopp, Jacinta Lee Johnson

**Affiliations:** 1 College of Medicine and Public Health, Flinders University, Bedford Park, SA 5042, Australia; smok0007@flinders.edu.au; 2 Southern Adelaide Local Health Network, Bedford Park, SA 5042, Australia; benkirsopp@gmail.com; 3 UniSA Clinical & Health Sciences, University of South Australia, Adelaide, SA 5001, Australia; 4 SA Pharmacy, SA Health, Adelaide, SA 5001, Australia

**Keywords:** opioid, inpatient, hospital, medication safety, respiratory depression, coroner’s inquest

## Abstract

Although opioids are the cornerstone of moderate-to-severe acute pain management they are appropriately recognised as high-risk medicines. Patient and health service delivery factors can contribute to an increased risk of death associated with excessive sedation and respiratory impairment. Despite increasing awareness of opioid-induced ventilation impairment (OIVI), no reliable method consistently identifies individual characteristics and factors that increase mortality risk due to respiratory depression events. This study assessed similarities in available coronial inquest cases reviewing opioid-related deaths in Australian hospitals from 2010 to 2020. Cases included for review were in-hospital deaths that identified patient factors, clinical errors and service delivery factors that resulted in opioid therapy contributing to the death. Of the 2879 coroner’s inquest reports reviewed across six Australian states, 15 met the criteria for inclusion. Coroner’s inquest reports were analysed qualitatively to identify common themes, contributing patient and service delivery factors and recommendations. Descriptive statistics were used to summarise shared features between cases. All cases included had at least one, but often more, service delivery factors contributing to the death, including insufficient observations, prescribing/administration error, poor escalation and reduced communication. Wider awareness of the individual characteristics that pose increased risk of OIVI, greater uptake of formal, evidence-based pain management guidelines and improved documentation and observations may reduce OIVI mortality rates.

## 1. Introduction

While opioid medications provide a cornerstone for the management of acute moderate-to-severe pain, their ability to depress the central nervous system is well known. There are many adverse effects linked to opioid use ranging from side effects of nausea, vomiting and confusion to excessive sedation and respiratory depression potentially leading to increased morbidity and mortality. There are many terms used interchangeably when referring to “respiratory depression” including opioid-induced respiratory depression (ORID), opioid induced ventilatory impairment (OIVI) and opioid-related adverse events [1,2,3,4,5,6,7,8,9]. 

The Australian and New Zealand College of Anaesthetists and Faculty of Pain Management recommends OIVI as the most appropriate description, encompassing opioid-induced central respiratory depression (decreased respiratory drive), decreased level of consciousness (sedation) and upper airway obstruction [5]. Culminating in decreased ventilation and increased arterial carbon dioxide levels. The combination of hypoxia and hypercapnia commonly results in tachycardia and hypotension, before potential cardiorespiratory arrest, hypoxic encephalopathy and death if left untreated [10]. 

Challenges exist when attempting to study the prevalence of OIVI with varying definitions, dependent on outcome measures compared. A systemic literature review of postoperative patients demonstrated an incidence ranging from 0.04% when naloxone use was the outcome of interest, up to 41% if assessed by presence of hypoxaemia or bradypnoea [7]. Overall, the finding was that 0.5% of 841,424 total cases met the varying criteria for rescue management [7]. In another study, Jungquist et al. reported the incidence of opioid-induced respiratory depression in sentinel events to be <2% of all patients in hospital while on opioids [8]. Ramachandran et al. reported an incidence of 0.038% when measuring sudden-onset life-threatening respiratory events by studying patients found unresponsive while on analgesic therapy [11]. This has parallels with interpreting OIVI in the context of coroners’ reports as the measurement criteria is death.

It is clear that while there are benefits to providing adequate pain management with opioid analgesia, it comes with associated risks and possible mortality. There are lessons to be learnt from the cases that progress from “respiratory depression” to death. In Australia, the state coroners inquire into unexpected deaths in hospitals, with thorough investigations involving staff, family and evidence from expert witnesses regarding the surrounding circumstances. This gives a unique opportunity to evaluate such cases in the context of collating common themes associated with mortality and assessing the recommendations made by the coroner to minimise the risk of further opioid-related deaths. 

The purpose of this review was to investigate in-hospital, opioid-related deaths across Australia, utilising publicly accessible coroner’s case reports. This review aims to identify in-hospital opioid-related deaths from which to gather similarities and lessons learnt to reduce opioid-related harm.

## 2. Materials and Methods 

In May 2020, a retrospective review of publicly available coroner’s inquest case reports for deaths in Australia between 2010 and 2020 was conducted. Deaths occurring in the Australian Capital Territory and Victoria were excluded due to lack of public access and document word search ability, respectively. NSW and WA only had published records available from 2012 to 2020.

An excel spreadsheet was constructed with four columns. The first column had the particular state, then the relevant year under that (beginning at 2020), with each name pertaining to the inquest below that. A new year was added to separate groups of names. Each name had a column space next to it, from left to right, for whether an opioid was involved, a comment on the manner of death and a highlight space to insert a colour if the case was to be “flagged” for a comprehensive review. An example of the structure is attached in the Appendix A and Appendix B with de-identified data. 

The coroner’s inquest reports can be accessed via the respective state government websites, which are linked in the Appendix A and Appendix B. Cases were opened and initially assessed for opioid involvement in five areas and in the following order for any mention of “opioid”, “pain”, “analgesia” or any opioid or opioid-related drug; key-/catchwords, manner and cause of death, case background and toxicology report. If any of these phrases were identified in those subsections, it was immediately flagged for a more comprehensive review later. If within the manner or cause of death, there was reference to “respiratory depression”, “drug toxicity”, “hypoxic brain injury” or “aspiration pneumonia”, these were reviewed at the time to assess whether opioids were involved in the care. 

During this primary review, cases were excluded if the coroners themselves ruled, based upon all the available evidence, that opioids administered were not directly implicated in causing, hastening or significantly contributing to death. Cases were excluded if the subject was in custody at the time or if the inquest was a part of routine investigations for those under inpatient treatment orders. Cases were also excluded where opioid information was scarce and could not be determined as an important or relevant part of their pharmacological care. The remaining cases were then reviewed under the criteria that the death must have occurred in a hospital or affiliated treatment facility, such as in drug and alcohol rehabilitation units. Cases were then excluded from further review if the opioid or other drugs considered to contribute to the death were not administered/prescribed by hospital staff (self-administered without knowledge of staff, this does not include patient-controlled analgesia). 

The complete coroner’s inquest reports that met inclusion criteria were analysed qualitatively to identify common themes, contributing factors and recommendations, with a focus on patient factors, avoidable clinical errors and health service delivery factors. Data were extracted using a standardised data collection spreadsheet. Descriptive statistics were used to summarise features shared between cases. 

## 3. Results

From 2010 to 2020, across South Australia, New South Wales, Northern Territory, Queensland, Western Australia and Tasmania, there were 2879 coroner’s inquest reports available for public view. Of these, 132 (4.58%) involved opioids. Of cases where opioids were involved, the vast majority 94 cases (71.21%) occurred in the community, 10 (7.58%) occurred under police custody and five (3.79%) in aged-care facilities, these were excluded. A further three cases were excluded as the patients had self-administered additional opioids whilst admitted in hospital and an additional case was excluded after medications were self-administered at home prior to death once admitted to hospital. Of the remaining 19 cases, 4 were reviewed and discussed by all 3 authors, with a consensus that the opioid involvement was ambiguous as a cause of death and that other factors were more important in the coroner’s consideration, leading to minimal learning points or recommendations surrounding the safe use of opioid analgesia. Thus, a total of 15 met the inclusion criteria for analysis. 

A summary of the patient demographics and comorbidities for the cases included in the review is presented in Table 1. Table 2 summarises the presenting complaints and preceding events, prior to the OIVI event for each case.

Across the 15 cases included for detailed review and analysis, the age range was 23—88 years old, with a mean age of 55.7 (SD: 18.2) years and a median age of 54 years. Nine (60%) cases were male, and six (40%) were female. Nine (60%) cases were situated from the public hospital sector, and six (40%) were from private hospitals or associated facilities. There was a relatively even split between medical (40%), surgical (33.33%), mental health (13.33%), drug and alcohol (6.67%) and Emergency Department/Short Stay Unit (6.66%). From medical admissions, three (50%) occurred on general medical wards, two (33.33%) being palliative and one (16.67%) being neurology. Orthopaedics comprised 80% of surgical cases, with general surgery comprising 20%.

### 3.1. Time of Event

Between all cases, the incidence of the event appears skewed towards the first day (33.33%). For patients undergoing a procedure, the event was most likely to occur in the first 24 hours (60%) following the operation, with the remaining occurring on day 2 after the operation (40%). The length of time to event after surgery ranged between 8 and 48 h, with a mean time to event of 23.6 h and a standard deviation of 15.7 h. 

### 3.2. Patient Comorbidities

The patients in six (40%) cases were reported to be overweight. Of the four (26.67%) who had a BMI reported, all were in the obese range with BMI greater than 38.1. A further two more (13.33%) were described as overweight in the reports without BMI documented. 

Five (33.33%) cases had confirmed (three) or suspected (two) obstructive sleep apnoea. Six (40%) had some type of heart disease (including coronary artery or ischaemic heart disease and heart failure) and four (26.67%) had confirmed diabetes mellitus. Only one case (6.67%) reported either chronic lung disease (emphysema) or chronic kidney disease.

### 3.3. Contributing Opioids and Other Sedatives

Morphine was involved in seven cases (46.67%), while fentanyl was implicated in four cases (26.67%). 

Of these six cases, three involved parenteral administration, two involved transdermal administration and one involved both parenteral and transdermal routes, as the transdermal opioid prescribed was intended for another patient. Pethidine was involved in two cases (13.33%). Two cases (13.33%) involved oxycodone immediate release and three (20%) involved oxycodone modified release, with these cases (20%) all involving use of a combination of long- and short-acting opioids. Hydromorphone was involved in two cases (13.33%), one being given to the wrong patient and one having an incorrect dose administered. 

Two (13.33%) of the cases involved patient-controlled analgesia (PCA), with a further one having PCA disconnected (indwelling venous catheter tissued) in the hours prior to the event occurring. 

Methadone was implicated in three (20%) cases; in two cases, methadone was indicated for opioid substitution therapy (OST) and, in one case (6.67%), it was prescribed for the management of acute pain. A further case (6.67%) involved buprenorphine prescribed for OST. All three cases pertaining to OST involved a combination of opioids of benzodiazepines and/or antipsychotics.

Benzodiazepines were documented to have been administered in five of the cases (33.33%) with a further case reporting a benzodiazepine in the blood toxicology report. Gabapentinoids and antipsychotics were both prescribed in three cases (20%), while antihistamines and sedating antidepressants were prescribed in two cases (13.33%). Overall, eight (53.33%) of the cases had a co-prescribed sedating medication, with five (33.33%) of these cases involving a combination of one or more of the above medications. 

Table 3 provides a comprehensive description of the opioids used (including route and dose) and co-prescribed sedating agents involved in the included cases.

### 3.4. Health Service Delivery Factors

Four broad categories of service delivery factors contributed to the opioid-related deaths in the cases emerged upon review. These related to prescribing or administration issues, observations, escalation procedures and communication. Table 4 contains a summary of the service delivery factor subcategories and which cases had evidence of these embedded within. 

Amongst the 15 cases, 12 (80%) had a prescribing or administration issue. Seven cases (46.67%) reported uncertainty presented by the coroner, colleagues or expert witnesses surrounding the appropriateness of the dose prescribed. Five cases (33.33%) identified local policies or procedures that required updating. Four cases (26.67%) included a scenario where there was a lack of awareness surrounding being prescribed multiple opioids together or in combination with sedative medications such as benzodiazepines or antihistamines. Two cases (13.33%) involved giving the wrong patient the wrong medication, one being prescribed incorrectly and one administered incorrectly. Two further cases (13.33%) involved a dose calculation error, one both in prescribing and administering the opioid. 

Twelve cases (80%) reported issues with observations. Eight cases (53.33%) highlighted instances of insufficient monitoring or observations. Six cases (40%) reported instances of insufficient documentation of observations and vital signs. Six cases (40%) included failure to increase the level of observations once deterioration or concern was identified. 

Eleven cases (73.33%) involved issues with escalation procedures. Nine cases (60%) included inadequate knowledge from hospital staff surrounding early clinical signs of deterioration. Six cases (40%) included a failure to escalate appropriately once deterioration was identified. Eight cases (53.33%) failed to involve an anaesthetist or seek out an expert pain management review when required. 

Ten cases (66.67%) involved communication issues. Six cases (40%) included a lack of clear instructions to other staff. Five cases (33.33%) consisted of instances of inadequate notification of clinical deterioration or other concerns. Five cases (33.33%) consisted of poor communication amongst the health professionals working at the time. Three cases (20%) involved poor handover practices, and another three included consisted of instances of poor communication about patients’ contributing risk factors for respiratory depression. 

Table 5 summarises recommendations and themes for improvement made by the coroners, stratified into categories.

## 4. Discussion

This paper summarises the deaths of 15 individuals, in which opioid medications were implicated. These cases represent a heterogenous group of patients; however, they share similar thematic undercurrents. While this study is not powered to draw statistically significant conclusions, it can be used in conjunction with similar studies worldwide to demonstrate important factors that need to be considered when prescribing and caring for patients receiving opioid medications to improve safety [2,4,7,8,12,13]. 

The period immediately following surgery appears to represent a time of elevated risk for OIVI. From the literature, proximity to surgery was overwhelmingly involved with greater than 85% of all deaths occurring within 24 h of surgery [9,12]. This correlates somewhat with our data, with 60% of the events occurring within the first 24 h after the procedure and the remaining postsurgical events occurring the following day. This could be due to medical professionals’ awareness of increased mortality within 24 h after surgery and, therefore, compensating by being hypervigilant. 

Studies have consistently shown statistically significant increased risk of postoperative OIVI in patients with pre-existing cardiac and pulmonary disease, as well as those with obstructive sleep apnoea (OSA) [7]. This was partially reflected in our cohort, particularly for cardiac disease and OSA, with 40% and 33.33% of all cases having these comorbidities, respectively. This increased to 60% of all surgical cases having either, with 40% of surgical cases having both OSA and cardiac disease. Only one patient, who was a postoperative case, had concurrent pulmonary disease. While only three of the five OSA cases had been formally diagnosed, they were included as the coroners had deemed them likely to have undiagnosed OSA. These patients with undiagnosed OSA had a BMI of 38 or above. In one study, 59% of chronic opioid users had evidence of previously undiagnosed OSA when undergoing polysomnography [14]. Thus, given the associated risk, timely screening and testing for OSA, particularly in cases with high a BMI could improve safety when prescribing opioids. In a study by Rao and Khanna, it has been noted that while there are assessment tools for OSA and respiratory impairment, such as the STOP-Bang questionnaire, ARISCAT and SPORC, each has its limitations, and a method to stratify all patients who may develop OIVI remains elusive [15].

The effect of OIVI is increased following co-administration of benzodiazepines [16]. Overdose deaths in patients co-prescribed opioids and benzodiazepines has been shown to be 9–10 times that of patients prescribed opioids alone [17]. The combination of opioids and gabapentinoids (gabapentin and pregabalin) have also been shown to have a lesser, but ongoing additional risk on mortality when co-prescribed with opioids [18]. Amongst our findings, benzodiazepines were involved in six (40%) of cases. Admittedly, only one of these cases listed the benzodiazepine as the only co-prescribed sedating agent, the others contained a mix of antipsychotics (three), a gabapentinoid (one) or an antihistamine (one), highlighting the need to review the total sedative load to assess and minimise the risk of OIVI.

In response to these cases, the coroners made recommendations promoting broader awareness of the inherent risk of respiratory depression in patients receiving opioid analgesia. Recommendations were made surrounding opioid prescribing as concerns relating to the prescribing process were raised in 80% of the cases. These recommendations encompassed dose, dosing intervals and routes, such as transdermal fentanyl patch prescription being limited to pain treatment specialists and not used to manage acute pain. Additionally, prescribers should be aware of previous opioid use (or naivety) and concurrent sedating medications as discussed above. Dependent on the hospital or health system, recommendations to improve safety have involved; standardising protocols for age-appropriate opioid prescribing, increased clinical education, availability of specialised acute pain teams, new acute pain charts and protocols implemented to improve monitoring and detect deterioration. A study by Lee, Caplan and Stephens found that 97% of opioid-induced respiratory depression that resulted in death or severe brain injury could be prevented by adjustments in medication administration or enhanced monitoring [12]. 

Concern with level of observation was noted in 80% of cases, with insufficient monitoring highlighted in 53.33%. Monitoring for OIVI can include sedation score, respiratory rate and oxygen saturation. In a study monitoring postoperative oxygen saturations continuously, it was found that not only was reduced oxygen saturation common in those receiving opioids, with 20% of all patients experiencing SpO_2_ <90% for >10 min per hour, but that 90% of prolonged hypoxic events (SpO_2_ < 90% for ≥1 h) were frequently missed on routine (4 hourly) nursing reviews [19]. This was also highlighted in another study that showed a benefit of having continuous pulse oximetry with centralised alarms [20]. In our cohort, in many cases focus was placed on lack of nursing reviews assessing these factors; however, it has been previously shown that up to a third of OIVI events occur within an hour from the last medical review [12]. Moving forward, improved techniques of monitoring could be utilised to minimise the occurrence and risks associated with OIVI. Other electronic monitoring devices for high-risk patients, such as monitoring end-tidal CO_2_ and minute ventilation can detect events before SpO_2_ falls and OIVI becomes clinically evident [4,8].

Another common theme across the cases reviewed was inadequate recognition of signs of deterioration by clinical staff (occurred in 60% of cases) and failing to escalate or increase observations when deteriorations were detected (occurred in 33.33% of cases). General communication issues were highly prevalent, identified in 76.67% of our cohort. Some of the recommendations suggest increased use of guidelines/protocols, with structured systems for patient monitoring and availability of avenues for escalation when required. 

In future, novel opioid development could target the provision of potent analgesia whilst minimalising respiratory depression, utilising co-administration of respiratory stimulants potentially reducing OIVI events [21].

## 5. Limitations

It is important to note that the coroner’s reports are legal documents pertaining to the narratives surrounding an unsuspected death. They do not contain vivid or succinct medical notes, and itis not possible or wise to draw conclusions beyond those stated in the report. In many cases, particularly the monothematic cases such as where the incorrect doses were given, patient factors, details and other service delivery factors were absent. For instance, only one patient had documented renal disease; however, with many cases complicated by hypertension and heart disease, the incidence of this could be assumed to be much higher. Similarly, BMIs, smoking status, full medication lists and total opioid doses administered were incomplete. Another limitation of this study is its small sample size of 15 cases.

## 6. Conclusions

Wider awareness of the individual characteristics that pose an increased risk of OIVI, greater uptake of formal, evidence-based pain management guidelines, improved communication, observations and more appropriate escalation of care may reduce OIVI mortality rates. 

## Figures and Tables

**Table 1:** Coroner’s inquest report date, patient demographics and comorbidities.

Case	Year	State	Age	Gender	BMI	Comorbid Conditions	Smoking Status
**MC**	2016	QLD	33	M	Unknown, overweight	OSA, anxiety, previous viral meningitis	Unknown
**DP**	2012	QLD	45	M	40	OSA, T2DM, hypertension, osteoarthritis, previous wrist open reduction and internal fixation	Unknown
**JC**	2019	SA	79	F	Unknown	Heart failure (ischaemic + valvular)	Unknown
**SA**	2018	SA	53	M	Unknown	Chronic neck and back pain	Unknown
**JR**	2014	SA	54	M	41	Gastro-oesophageal reflux disorder	Unknown
**CP**	2013	SA	72	F	Unknown	Metastatic parotid acinic carcinoma, pelvic fracture	Unknown
**PL**	2018	NSW	54	M	Unknown	Coronary artery disease, hypertension, hypercholesterolaemia, OSA not using continuous positive airway pressure (CPAP) at home	Ex-smoker
**AM**	2016	NSW	88	F	Unknown	Heart failure, acute pulmonary oedema, prosthetic aortic valve replacement, chronic renal failure	Unknown
**W**	2018	NSW	38	M	Unknown	Opiate and alcohol use disorder	Unknown
**SO**	2019	WA	54	M	39	Schizophrenia, attention deficit hyperactivity disorder, narcolepsy, oculocutaneous albinism, achalasia, possible undiagnosed OSA	Unknown
**MJ**	2017	WA	59	F	38.4	T2DM, hypercholesterolaemia, angina, gastro-oesophageal reflux disorder, possible undiagnosed OSA, extensive surgical history, notable recent washout for right groin abscess	Yes
**GR**	2016	WA	23	M	Unknown, overweight	Schizophrenia	Yes
**TB**	2016	TAS	45	F	Unknown	End-stage metastatic cervical cancer, metastasis to liver and spine	Unknown
**EB**	2011	TAS	76	F	Unknown	Alcoholic pancreatitis (reformed), cholelithiasis, ischaemic heart disease, coronary artery bypass graph ×3, mitral valve repair, hypertension, T2DM, gout, depression	Unknown
**CK**	2011	TAS	79	M	Unknown	Atherosclerotic heart disease, hypertension, emphysema, T2DM, osteoarthritis	Unknown

Abbreviations: BMI = body mass index, F = female, M = male, OSA= obstructive sleep apnoea, T2DM = type 2 diabetes mellitus.

**Table 2:** Reason for admission, details of presenting complaint, death and events prior to opioid induced ventilatory impairment (OIVI).

Case	Reason for Admission	Public vs. Private Hospital	Admitted Under	Preceding Events	Time of Death	Cause of Death
**MC**	Severe occipital headache	Private	Medical (General)	Occipital headache, neck pain and stiffness without cause identified. Started pregabalin and slow- and fast-acting opioids, up-titrated as minimal effect. OSA not known during admit. Decreased oxygen saturation in Emergency to 79% after IV morphine, not acted upon. Nil overt narcotisation. Sleeping. Aspirated.	04:40 unresponsive, 05:17 deceased	Opioid toxicity causing central and respiratory depression and aspiration pneumonia
**DP**	Elective removal of left wrist place	Public	Surgical (Orthopaedic)	Elective removal of left open reduction and internal fixation. OSA unknown at time of operation. After the operation, PCA with background dosing started but background was ceased as drowsiness and a desaturation occurred. Pruritic and behaviourally agitated. Given promethazine and temazepam. Transferred wards, unresponsive. Eleven hours after procedure.	02:00 unresponsive, resuscitated, hypoxic encephalopathy, died four days later	Hypoxic brain injury from respiratory depression in context of OSA, morbid obesity, possible respiratory infection and administration of sedatives
**JC**	End-stage cardiac failure	Public	Medical	Admitted for management of end-stage cardiac failure with associated liver and renal failure. During admit, given hydromorphone intended for another patient. Deteriorated. Commenced on palliative care.	12:00 given dose, died nine days later.	Multi-organ failure as a result of IHD and valvular heart disease, urinary tract infection and the effects of hydromorphone
**SA**	Headache and right arm pain	Public	Medical (Neurology)	Week-long history of Horner’s syndrome and right arm radiculopathy. Admitted for analgesia and non-urgent MRI. Pain not settled with increasing analgesia prescribed. Drowsy, apnoeic despite inadequately controlled pain.	06:05 unresponsive, 06:45 deceased	Fentanyl and oxycodone toxicity
**JR**	Ankle surgery	Private	Surgical (Orthopaedic)	Admitted for elective ankle surgery. No high dependency unit capacity, on PCA, canula failed and given as required instead. Snoring. Hypoxic, bradypnoeic. Arrested as paramedics arrived for transfer to tertiary hospital. Fifteen hours after right ankle arthrodesis.	06:00 unresponsive, died four days later	Hypoxic ischaemic encephalopathy secondary to cardiac arrest contributed by opiate analgesia and morbid obesity
**CP**	Uncontrolled pain from pathological fractures	Private	Medical (Palliative Care)	Had intrathecal spinal catheter put in, then ceased and taken out, with the portal left in, in place of oral pain management. Spinal analgesia was reinstituted 5 days later, however, treating palliative care physician mistakenly prescribed medication for an epidural portal, which was inappropriate for the intrathecal portal already inserted.	11:30 unresponsive, 14:30 deceased	Intrathecal toxicity of bupivacaine and morphine
**PL**	Left anterior cruciate ligament reconstruction	Public	Surgical (Orthopaedic)	Underwent reconstruction of left knee. While patient PL was in recovery and next in operating theatre, anaesthetist charting electronically left the electronic medical record linked to incorrect patient PH, prescribing PL multiple opioids intended for the next patient. Were given, patient became sedated, aspirated. Eleven hours after procedure.	00:38 unresponsive, 00:56 deceased	Aspiration pneumonia caused by multiple drug toxicity, particularly fentanyl
**AM**	*C. difficile* colitis, faecal transplant	Private	Medical	Recurrent *C. difficile* colitis flare, admitted for a colonoscopy and faecal transplant. Ongoing symptoms despite, contracted pneumonia and respiratory failure. Prescribed hydromorphone for respiratory distress. Given once (as morphine), then on second dose, incorrect dose of hydromorphone given. Interpreted mg as mL, gave in mL, 10× overdose. Identified and reversed, remained unresponsive. Fifteen days after faecal transplant + colonoscopy.	12:30 unresponsive, 16:30 deceased	Combined effects of overdose of hydromorphone and complications of pneumonia + *C. difficile*. On background of heart and lung disease.
**W**	Opioid and alcohol withdrawal treatment	Public	Drug and alcohol	Alcohol and opioid withdrawal treatment. Started buprenorphine and diazepam prescribed by on-call doctor. Noted to have pinpoint pupils after second dose. Not escalated and given third dose. Somnolent.	15:40 unresponsive, 19:25 pronounced deceased	Respiratory depression most likely from excessive buprenorphine
**SO**	Manic episode schizoaffective disorder, under ITO	Public	Mental health	Receiving methadone in community, query compliance, given presumed regular dosing, also given multiple sedatives (zuclopenthixol 3 doses in 5 days), became sedated, thought to be asleep.	07:55 unresponsive, nil CPR commenced as rigor mortis present	Methadone toxicity
**MJ**	Wound dehiscence/cellulitis right groin wound	Private	Surgical (General)	Admitted for management of dehiscent right groin wound following previous abscess washout. Started on methadone for pain during stay. After theatre, started on fentanyl patch, due to other medication/PCA adverse effects. Vomited night after surgery and medications re-administered. Thirty-six hours after excision and examination of right groin.	00:20 unresponsive, 01:28 deceased	Opioid toxicity, predominantly fentanyl
**GR**	Chronic schizophrenia relapse,	Public	Mental health	Admitted for psychotic relapse for clozapine titration. Wanted to be restarted on methadone for pain. Prescribed as per “Next Step” by drug and alcohol service team. Noticed to be sedated and had refused observations.	14:10 unresponsive, 14:45 deceased	Combined drug toxicity—methadone major contributor
**TB**	Intractable pain from malignancies	Public	Medical (Palliative Care)	Admitted for palliative pain relief. Morphine dose for syringe driver miscalculated and administered. Given overnight, leading to bradypnoea, hypoxia, hypotension and decreased consciousness. Ceased and reversed morphine, given IV fluids and IV antibiotics but deteriorated.	17:10 deceased	Metastatic cervical cancer, bronchopneumonia, accelerated by morphine overdose
**EB**	Abdominal pain	Public	Emergency department/Short Stay Unit	Admitted for right upper quadrant pain, thought gallstones/pancreatitis. For review in morning. Staff shortages led to insufficient observations.	05:20 unresponsive, 05:36 deceased	Pancreatitis and IHD, likely contributed by pethidine
**CK**	Bilateral knee replacement	Private	Surgical (Orthopaedic)	Elective knee replacement, postsurgical pain treated by femoral nerve block and combination long and short opioids. Found sleeping without concerns of impending demise by doctor, not woken, half hour before found unresponsive. Forty-eight hours after bilateral knee replacement.	11:45 unresponsive, 12:10 deceased	Combined drug toxicity, recent surgical procedure/anaesthesia, IHD and emphysema.

Abbreviations: ITO = inpatient treatment order, IV = intravenous, OSA = obstructive sleep apnoea, PCA = patient-controlled analgesia.

**Table 3:** Opioids involved, route of administration, dose and co-prescribed sedating agents involved in included coroners’ cases.

Cases	Opioid Tolerance	Duration on Opioids in Hospital	Opioids (Route) and Dose Prescribed	Toxicology (If Available)	PCA/Nerve Block	Dose Administered in 24 h Preceding Death	Benzodiazepines	Gabapentinoids	Antihistamines	Antipsychotics or Antidepressants
**MC**	Naïve	54 h	Oxycodone MR (PO) 80 mg BD Up-titrated over admission, was increased and 1× dose 80 mg given Morphine PO 20–40 mg PRN	Morphine lethal range oxycodone above therapeutic, below toxic range Rest within normal range		200 mg morphine (PO) 120 mg oxycodone MR (PO) Over 54 h given 535–595 mg oral morphine equivalent	?diazepam (levels within blood, on in community, unsure in hospital)	Gabapentin		
**DP**	Tolerant	12 h	PCA: Morphine 2 mg/h background (ceased 5 h prior to event) with 10-minutely 2 mg PRN		Yes	10 mg IV morphine intra-op 110 µg IV fentanyl intra-op 25 mg IV morphine in recovery 2 mg/h background morphine PCA 2 mg morphine 10-minutely PRN PCA	Temazepam 20 mg		Promethazine 30 mg	
**JC**	Naïve	Single dose	None			16 mg hydromorphone PO				
**SA**	Codeine, previously tolerant to other opioids	4 days	Oxycodone MR (PO) 30 mg BD Oxycodone IR (PO) 10–20 mg QID PRN Fentanyl 75–150 µg (SUBCUT) 2 hourly unless sedation score < 2	Oxycodone 0.2 mg/L (therapeutic 0.02–0.05 mg/L) Fentanyl 4 µg/L (therapeutic level 0.6–3.9 mcg/L)		80 mg oxycodone MR (PO) Unsure exact PRN, in last 8.5 h; 20 mg oxycodone IR (PO) 450 µg fentanyl (SUBCUT)		Pregabalin		
**JR**	Naive	16 h	Morphine PCA ?rate/bolus Morphine IM/SUBCUT (if PCA fails) PRN		Initially yes, but canula failed	34 mg morphine PCA 10 mg morphine IM/SUBCUTDextropopoxyphene				
**CP**	Tolerant	20 days	Bolus doss of 5 mg morphine and 3 mL 0.5% bupivacaine twice daily, into epidural portal	Significant concentrations of morphine and bupivacaine in cerebral spinal fluid Therapeutic concentrations of hydromorphone, morphine, fentanyl, amitriptyline	Yes	5 mg bolus morphine intrathecal 3 mg 0.5% bupivacaine bolus intrathecal				Amitriptyline
**PL**	Naïve	12 h	Fentanyl PCA 60 mL 20 µg/mL Fentanyl (transdermal) 100 µg/h Oxycodone (PO) 5–10 mg 4-hourly PRN	Fentanyl 8 µg/L (potentially fatal range 3–28 µg/L) Ropivacaine 2.5 mg/L (possibly toxic)	Yes	Fentanyl (transdermal) 100 µg/h PCA ?amount used				
**AM**	Naïve	1 day ?hours	Hydromorphone 0.5 mg (SUBCUT) once only then reordered PRN			5 mg (0.5 mL of 10 mg/1 mL) (SUBCUT) hydromorphone 0.5 mg (SUBCUT) morphine				
**W**	Tolerant	12 h	Buprenorphine (SL) 4–8 mg initially 4–8 mg after 1.5 h 4–8 mg PRN for breakthrough			20 mg buprenorphine–4 mg initially, then 8 mg (×2)	Diazepam			Quetiapine Mirtazapine
**SO**	Previously tolerant to 80 mg methadone, query compliance in community	5 days	Methadone (PO) 40 mg mane, 30 mg nocte	Methadone 0.67 mg/L blood, 4.2 mg/L liver		70 mg methadone	Clonazepam Diazepam			Zuclopenthixol acetate Quetiapine Olanzapine
**MJ**	Some tolerance, although overestimated. As per general practitioner just prior to admit, nil prescribed	36 h (current regime) 10 days on methadone, 13 days on pethidine/codeine	Fentanyl (transdermal) 75 µg/h Methadone (PO) 5 mg BD Pethidine (?IM) 50 mg Codeine	Fentanyl 12 µg/L		75 µg/h fentanyl (transdermal) 5 mg methadone (PO) 50 mg pethidine (IM)		Pregabalin	Doxylamine	Amitriptyline 100 mg nocte
**GR**	Opioid tolerant, however, had lost level of tolerance	3 days	Methadone (PO) 50 mg daily	Methadone 0.38 mg/L		50 mg methadone	Diazepam Temazepam (not found in blood)			Chlorpromazine Clozapine Quetiapine
**TB**	Tolerant	1 day	Morphine (SUBCUT) 45 mg/h via syringe driver Fentanyl (IN) 50 µg once only	Reported in high lethal range for morphine, caveat tolerance and post-mortem distribution		496 mg morphine (SUBCUT) 50 µg fentanyl (IN)				
**EB**	Unknown, likely relatively naïve	6 h	Morphine IV 2.5–10 mg ?once only Pethidine 100 mg IM ?once only	Pethidine 0.7 mg/L Morphine 0.09 mg/L		4 × 2.5 mg IV morphine 100 mg pethidine IM	Temazepam 20 mg			
**CK**	Naive	48 h	Oxycodone MR (PO) 20 mg BD Morphine (IM) 10 mg once only Tramadol (IV) 100 mg PRN Oxycodone (PO) 20 mg three-hourly PRN	Tramadol 1.1 mg/L Oxycodone 0.1 mg/L Morphine 0.03 mg/mL Ropivacaine 46 mg/L	Femoral nerve catheter—ropivacaine	10 mg morphine IM 40 mg oxycodone MR PO 2 doses oxycodone IR (?dose)				

Doses included where available. A preceding “?” indicates reported uncertainty regarding that factor. Abbreviations: BD = twice daily, IM = intramuscular, IN = intranasal, IR = immediate release, IV = intravenous, MR = modified release, PCA= patient-controlled analgesia, PO = orally, PRN = when required, QID = four times a day, SUBCUT = subcutaneous.

**Table 4:** Common health service delivery factors identified across all included coroners’ cases and grouped into subthemes.

Service Delivery Factors	SO	MJ	GR	TB	EB	CK	MC	DP	W	PL	AM	JC *	SA	JR	CP
**Prescribing or Administration**															
Wrong dose given				●						●	●	●			●
Lack of awareness of the risks surrounding multiple opioids or sedative medications prescribed	●		●							●			●		
Uncertainty surrounding appropriateness of dose prescribed			●		●		●			●	●		●		●
Local policies/procedures/protocol requiring update	●				●	●							●	●	
**Observations**															
Insufficient frequency	●	●	●		●			●		●			●	●	
Insufficient documentation	●		●			●	●	●					●		
Failure to increase observation frequency once deterioration or concern was detected		●					●		●	●			●		●
**Escalation**															
Poor knowledge of early clinical signs of deterioration	●	●	●				●		●	●	●		●	●	
Failure to escalate appropriately once deterioration was present							●	●	●				●	●	●
Failure to involve anaesthetist/seek out expert pain review		●	●				●	●	●	●			●	●	
**Communication**															
Lack of notifying others about clinical deterioration or concerns		●	●				●						●	●	
Lack of clear instructions	●		●						●	●			●	●	
Poor handover practices							●			●			●		
Lack of communication about patients’ contributing risk factors for respiratory depression on opioids					●	●							●		
Poor communication between health professionals		●	●				●						●	●	

**Table 5:** Coroners’ recommendations and themes for improvement stratified into categories.

Recommendations/Themes for Improvement	SO	MJ	GR	TB	EB	CK	MC	DP	W	PL	AM	JC	SA	JR	CP
**Education**															
Regarding opioid prescribing and the risks associated			●	●			●		●	●			●	●	
Surrounding frequency and the reason for observations					●		●	●	●	●			●		
Identifying warning signs of deterioration							●	●	●	●			●	●	
Ability to treat and escalate as appropriate							●		●	●	●		●		
**Local protocol**															
Handover practices							●			●			●		
Anaesthetic/pain specialist assessment								●		●				●	
Introduction of new policy or amendment of current policy surrounding observations and documentation	●	●	●				●		●				●		●
Implementation of new escalation procedures							●		●				●		
Increase in level of staffing			●		●					●			●	●	
Amendment of hospital clinical practice guidelines/charts/protocols	●				●	●	●	●	●	●			●	●	
Review of pharmaceutical protocols				●						●					
**Broader policies**															
Department of Health codes/protocols/procedures		●	●							●	●				
Review of Department of Health directives											●				
Review and discussions by a specialty college (College of Anaesthetists and Pain Medicine) surrounding prescribing or dosing practices								●							
Amendment of specific state clinical guidelines									●		●		●		

**Figure A1:**
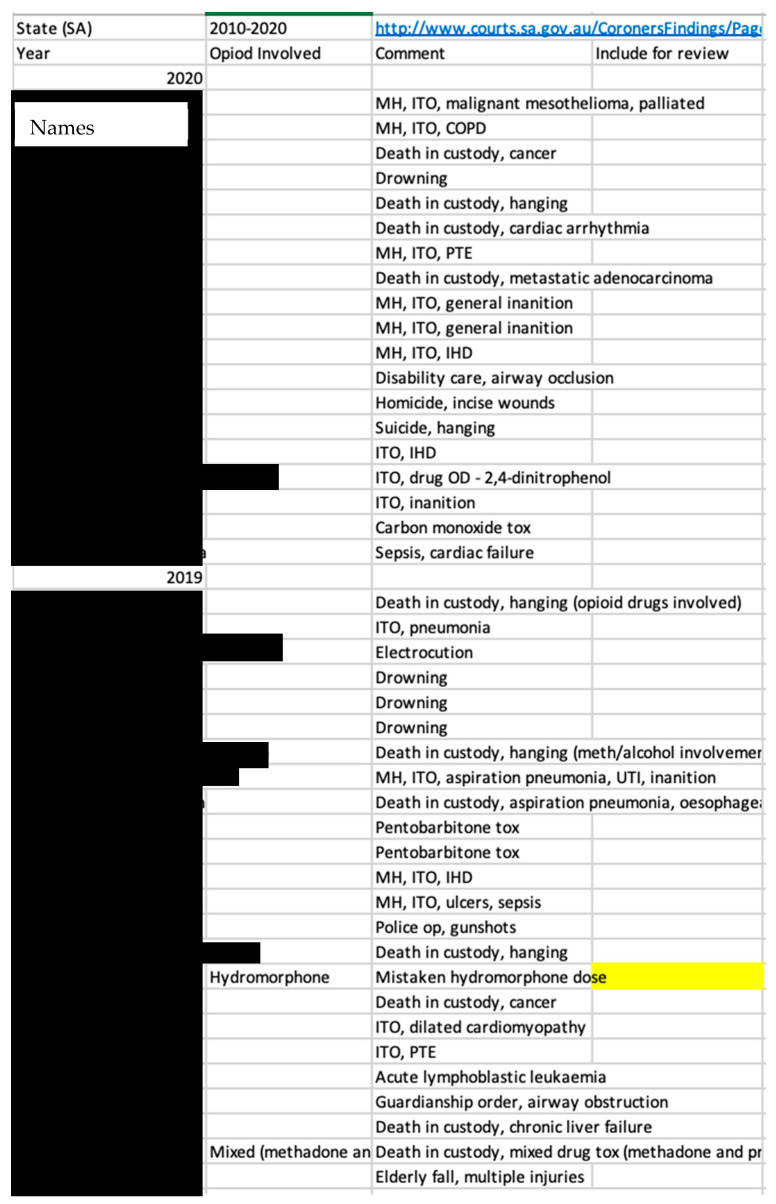
Example of the excel spreadsheet design used to track coroner’s cases to include for review. Below, the URL’s for websites used to access public coroner’s reports.

## Appendix B

**Table A1:** References for Coroner’s Reports for each State/Territory.

State/Territory	Coroner’s Reports
The State of Queensland (Queensland Courts)	https://www.courts.qld.gov.au/courts/coroners-court/findings (accessed on 15 May 2020)
Government of New South Wales (Coroners Court)	http://www.coroners.justice.nsw.gov.au/Pages/findings.aspx (accessed on 15 May 2020)
Government of Tasmania (Magistrates Court of Tasmania—Coronial Division).	https://www.magistratescourt.tas.gov.au/about_us/coroners (accessed on 15 May 2020)
Government of South Australia, Courts Administration Authority of South Australia (Coroners Court)	http://www.courts.sa.gov.au/CoronersFindings/Pages/default.aspx (accessed on 15 May 2020)
Northern Territory Government of Australia (Department of Attorney-General and Justice)	https://justice.nt.gov.au/attorney-general-and-justice/courts/coroners-findings (accessed on 15 May 2020)
Government of Western Australia (Coroner’s Court of Western Australia)	https://www.coronerscourt.wa.gov.au/I/inquest_findings.aspx?uid=6256-4150-5-7479 (accessed on 15 May 2020)
